# Lichen Extracts from Cetrarioid Clade Provide Neuroprotection against Hydrogen Peroxide-Induced Oxidative Stress

**DOI:** 10.3390/molecules27196520

**Published:** 2022-10-02

**Authors:** Isabel Ureña-Vacas, Elena González-Burgos, Pradeep Kumar Divakar, María Pilar Gómez-Serranillos

**Affiliations:** Department of Pharmacology, Pharmacognosy and Botany, Faculty of Pharmacy, Universidad Complutense de Madrid, Plaza Ramón y Cajal s/n, Ciudad Universitaria, 28040 Madrid, Spain

**Keywords:** lichens, neuroprotection, cetrarioid clade, oxidative stress

## Abstract

Oxidative stress is involved in the pathophysiology of many neurodegenerative diseases. Lichens have antioxidant properties attributed to their own secondary metabolites with phenol groups. Very few studies delve into the protective capacity of lichens based on their antioxidant properties and their action mechanism. The present study evaluates the neuroprotective role of *Dactylina arctica*, *Nephromopsis stracheyi*, *Tuckermannopsis americana* and *Vulpicida pinastri* methanol extracts in a hydrogen peroxide (H_2_O_2_) oxidative stress model in neuroblastoma cell line “SH-SY5Y cells”. Cells were pretreated with different concentrations of lichen extracts (24 h) before H_2_O_2_ (250 µM, 1 h). Our results showed that *D. arctica* (10 µg/mL), *N. stracheyi* (25 µg/mL), *T. americana* (50 µg/mL) and *V. pinastri* (5 µg/mL) prevented cell death and morphological changes. Moreover, these lichens significantly inhibited reactive oxygen species (ROS) production and lipid peroxidation and increased superoxide dismutase (SOD) and catalase (CAT) activities and glutathione (GSH) levels. Furthermore, they attenuated mitochondrial membrane potential decline and calcium homeostasis disruption. Finally, high-performance liquid chromatography (HPLC) analysis revealed that the secondary metabolites were gyrophoric acid and lecanoric acid in *D. artica*, usnic acid, pinastric acid and vulpinic acid in *V. pinastri*, and alectoronic acid in *T. americana*. In conclusion, *D. arctica* and *V. pinastri* are the most promising lichens to prevent and to treat oxidative stress-related neurodegenerative diseases.

## 1. Introduction

Oxidative stress is an imbalance in cellular redox homeostasis caused by ROS overproduction and/or antioxidant system dysfunction. The brain is particularly susceptible to oxidative stress and this process is increased with aging. The brain consumes almost 20% of the total basal oxygen, it is rich in polyunsaturated *n*-3 fatty acids and redox-active transition metals, it has a low endogenous antioxidant defense (i.e., catalase), and neurotransmitters such as dopamine can auto-oxidize leading to free radicals [1,2]. Moreover, the mitochondrial electron transport chain consumes around 98% of oxygen and the residual oxygen is converted into radical superoxide (O_2_^•−^) and the non-radical oxidant H_2_O_2_; excessive mitochondrial-derived ROS accumulation can lead to mitochondrial dysfunction [3]. Furthermore, hydrogen peroxide, the major redox metabolite, diffuses across membranes by water channels, oxidizes and damages biomolecules [4,5]. The main mechanism underlying the neurotoxic effects of hydrogen peroxide occurs through Fenton’s reaction. In this reaction, ferrous iron and hydrogen peroxide react to yield hydroxyl radical. Hydroxyl radical is the most deleterious free radical, reacting with macromolecules by hydroxyl addition and hydrogen abstraction [6]. The altered cellular redox homeostasis causes oxidative injury to lipids, proteins, and DNA and modifications in cellular function that finally contribute to cell death mainly by apoptosis [3]. Oxidative stress is involved as a major pathophysiologic mechanism of age-related neurodegenerative diseases such as Parkinson’s disease, Alzheimer’s disease, and amyotrophic lateral sclerosis [7].

Antioxidants modulate the redox state of cells through single electron transfer (SET), hydrogen atom transfer (HAT), transition metals chelation and the up-regulation of enzymatic and non-enzymatic antioxidants. Antioxidants are potentially beneficial in the prevention and treatment of central nervous system pathologies associated with oxidative stress and constitute one of the most promising therapeutical strategies [8].

Lichens have aroused great pharmacological interest in recent years because they produce compounds unique to these species. These bioactive compounds are primarily phenol derivatives such as dibenzofurans, depsidones and depsides. Lichen extracts and their secondary metabolites have shown an interesting antioxidant activity [8,9]. However, studies focusing on therapeutic and protective strategy based on the antioxidant ability of lichens are very limited [9].

Recently, in previous studies of this group, the antioxidant activity of lichen extracts from the cetrarioid clade was evaluated using different in vitro methods (1,1-Diphenyl-2-picrylhydrazyl (DPPH), oxygen radical absorbance capacity (ORAC) and ferric-reducing antioxidant power (FRAP) assays) and multivariate statistical techniques. This study revealed that the lichen species *Dactylina arctica* (Hook) Nyl., *Nephromopsis stracheyi* (C. Bab.) Müll. Arg., *Tuckermannopsis americana* (Sprengel) Hale, and *Vulpicida pinastri* (Scop.) J.-E. Mattsson & M. J. Lai. were the ones with the highest antioxidant capacities [10].

The aim of the present work is to evaluate for the first time the neuroprotective activity, based on antioxidant properties, of the methanol lichen extracts *Dactylina arctica*, *Nephromopsis stracheyi, Tuckermannopsis americana* and *Vulpicida pinastri* in a hydrogen peroxide-induced oxidative stress model in a neuroblastoma cell line. 

## 2. Results

### 2.1. Lichen Extracts from Cetrarioid Clade Promoted Neuronal Survival after H_2_O_2_-Induced Oxidative Stress

**Figure 1:**
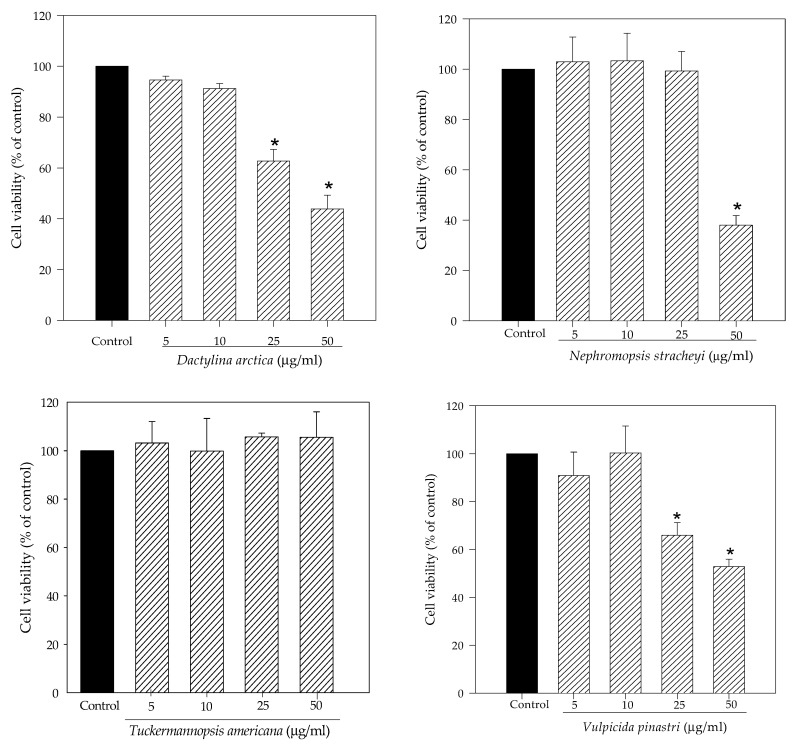
Effect of methanol lichen extracts of cetrarioid clade on cell viability. SH-SY5Y cells were treated with different concentrations of extracts from 5 to 50 μg/mL for 24 h. Cell viability was determined using MTT assay. Results are expressed as mean ± standard deviation (SD) (triplicate experiments). * *p* < 0.05 versus control.

Initially, we evaluated the effect of the methanol extracts of the lichens *Dactylina arctica*, *Nephromopsis stracheyi, Tuckermannopsis americana* and *Vulpicida pinastri* on the human neuroblastoma SH-SY5Y cell viability using 3-[4,5-dimethylthiazol-2-yl]-2,5 diphenyl tetrazolium bromide (MTT) assay. As shown in Figure 1, *T. americana* did not cause cytotoxicity at any assayed concentrations. On the other hand, *N. stracheyi* significantly reduced cell viability at 50 µg/mL (38% of cell viability) whereas *D. arctica* and *V. pinastri* affected cell viability at 25 µg/mL (62.7% and 65.9% of cell viability, respectively) and at 50 µg/mL (43.9% and 52.8% of cell viability, respectively). 

**Figure 2:**
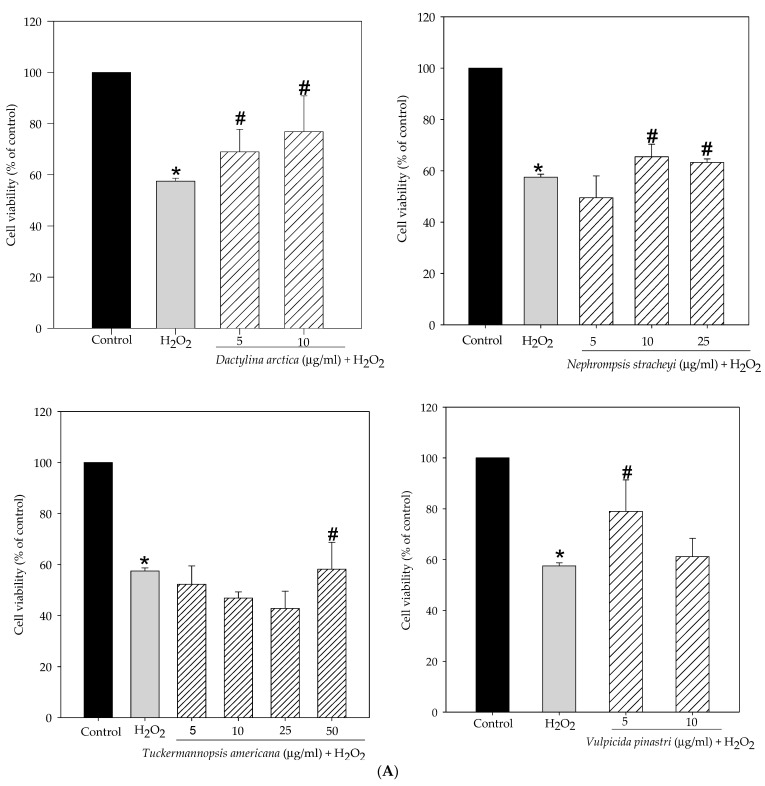

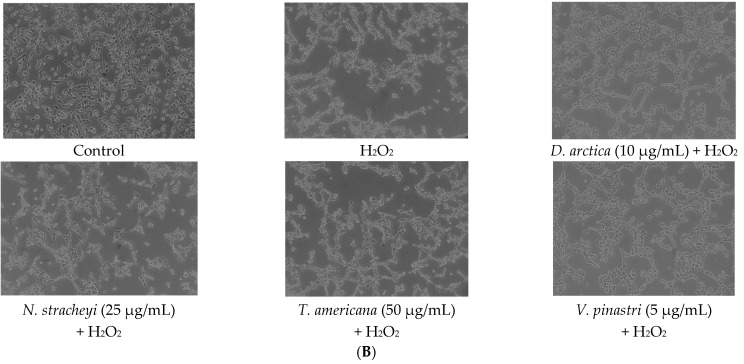
(**A**) Effect of methanol lichen extracts of cetrarioid clade on cytoprotection in stress oxidative models. SH-SY5Y cells were pretreated with non-cytotoxic concentrations of lichens for 24 h before H_2_O_2_ (250 µM, 1 h). Cell viability was determined using MTT assay. Results were expressed as mean ± SD (triplicate experiments). * *p* < 0.01 versus control; # *p* < 0.01 versus H_2_O_2_. (**B**) SH-SY5Y cells morphology after treatments.

Next, we investigated the potential protective effect of non-toxic concentrations of lichen extracts against hydrogen peroxide-induced oxidative stress. Figure 2A demonstrates that cell viability of the 250 µM for 1 h H_2_O_2_-treated SH-SY5Y cells significantly decreased by 57.5% compared to control cells (100%). However, pretreatments with methanol lichen extracts of cetrarioid clade promoted neuronal survival compared to hydrogen peroxide-treated cells. With 24 h pretreatment, the percentage of cell viability was increased over 68.9% and 76.8% for *D. arctica* at 5 and 10 µg/mL, respectively, over 65.4% and 63.2% for *N. stracheyi* at 10 and 25 µg/mL, respectively, 58.2% for *T. americana* at 50 µg/mL, and 78.9% for *V. pinastri* at 5 µg/mL. Therefore, we chose the most protective concentrations of each lichen extract to delve into the protective mechanism of these extracts and identify which of them is the most active. Hence, the maximum cell viability protection was 5 µg/mL for *V. pinastri*, 10 µg/mL for *D. arctica* and *N. stracheyi* and 50 µg/mL for *T. americana*. 

Figure 2B showed the effect of the most protective lichen extracts on cell morphology. Hydrogen peroxide (250 µM for 1 h) caused morphological changes toward a cellular SH-SY5Y rounding. By contrast, lichen extracts improved morphological changes of neuroblastoma cells as shown in the presence of cellular projections.

### 2.2. Lichen Extracts from Cetrarioid Clade Reduced ROS Production after H_2_O_2_-Induced Oxidative Stress

**Figure 3:**
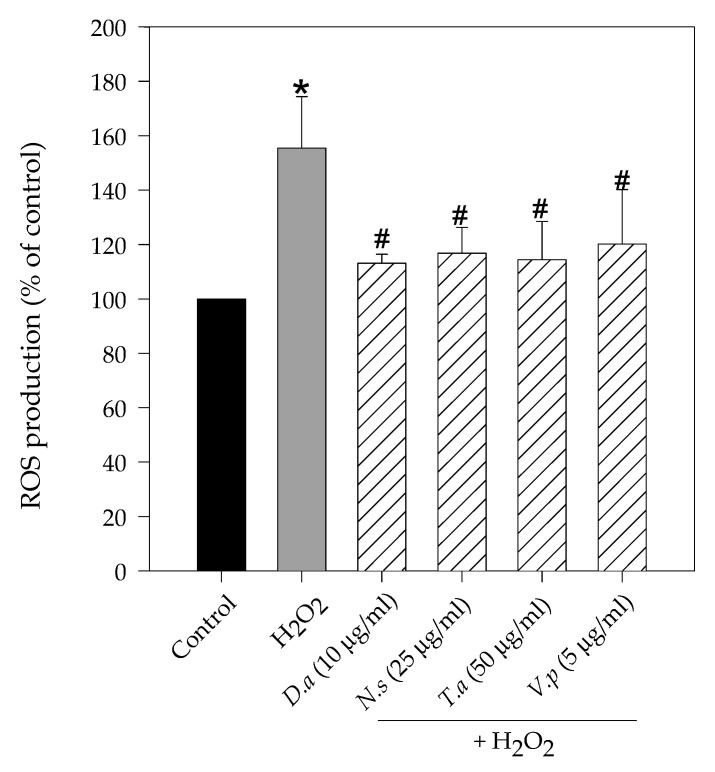
Effect of methanol lichen extracts of cetrarioid clade on intracellular ROS production. SH-SY5Y cells were pretreated with *D. arctica* (*D.a*), *N. stracheyi* (*N.s.*), *T. americana* (*T.a*) and *V. pinastri* (*V.p*) for 24 h before H_2_O_2_ (250 µM, 1 h). The levels of intracellular ROS production were measured using dichlorodihydrofluorescein diacetate (DCFH-DA) method. Results are expressed as mean ± SD (triplicate experiments). * *p* < 0.01 versus control; # *p* < 0.01 versus H_2_O_2_.

Figure 3 revealed a significant increase in intracellular ROS production when SH-SY5Y cells were treated with hydrogen peroxide. 1 h treatment with H_2_O_2_ at 250 µM enhanced ROS generation by 155.5% compared to control cells (100%). On the other hand, 24 h pretreatments with methanol lichen extracts significantly reduced ROS production. In particular, the highest reduction was shown by *D. arctica* (42.4% of reduction compared to hydrogen peroxide treatment), followed by *T. americana* (41.1% of reduction versus H_2_O_2_), *N. stracheyi* (38.7% of reduction versus H_2_O_2_) and *V. pinastri* (35.3% of reduction versus H_2_O_2_).

### 2.3. Lichen Extracts from Cetrarioid Clade Improved Oxidative Stress Markers and Antioxidant Enzyme Activity

**Figure 4:**
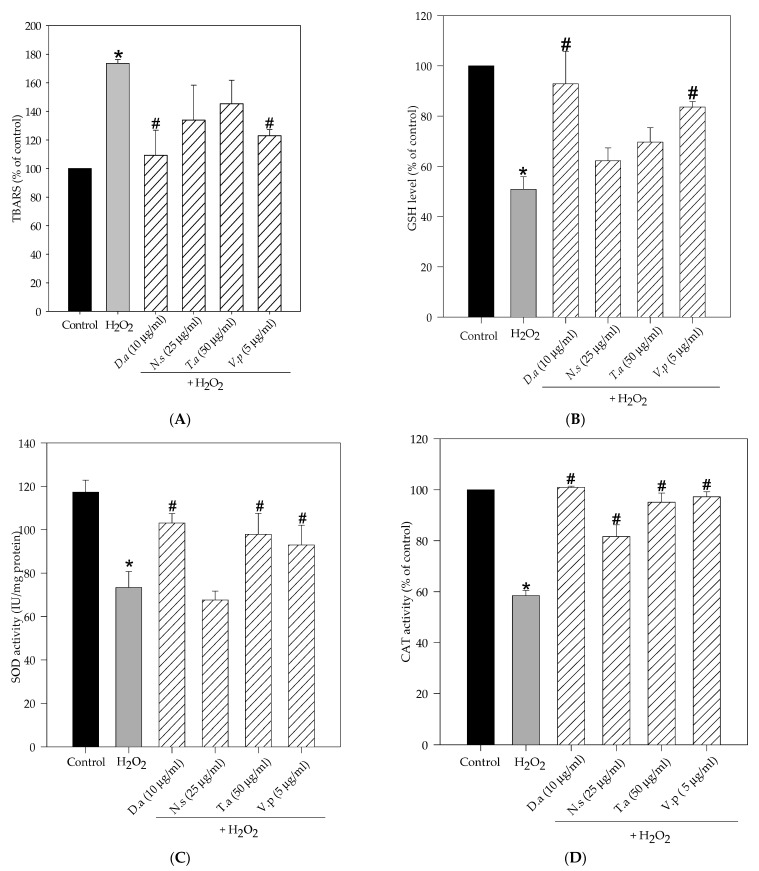
Lichen extracts from cetrarioid clade improved oxidative stress markers and antioxidant enzyme activity. SH-SY5Y cells were pretreated with *Dactylina arctica* (*D.a*), *Nephromopsis stracheyi* (*N.s*), *Tuckermannopsis americana* (*T.a*) and *Vulpicida pinastri* (*V.p*) for 24 h before H_2_O_2_ (250 µM, 1 h). (**A**) Lipid peroxidation, (**B**) GSH levels and (**C**) SOD activity, (**D**) CAT activity. Results are expressed as mean ± SD (triplicate experiments). * *p* < 0.05 versus control; # *p* < 0.05 versus H_2_O_2_.

Exposure to hydrogen peroxide significantly increased thiobarbituric acid reactive substances (TBARS) levels (174%), impaired GSH content (51%) and reduced SOD (68%) and CAT activity (58.5%) in SH-SY5Y cells compared to control cells (100%) (Figure 4). However, pretreatments with methanol lichen extracts of cetrarioid clade exhibited noteworthy protection against hydrogen peroxide-induced oxidative injury by improving the antioxidant status. Hence, *D. arctica* and *V. pinastri* significantly reduced lipid peroxidation levels (109% and 122%, respectively), restored GSH content (93% and 83.5%, respectively) and increased SOD activity (96% and 87%, respectively). Moreover, *T. americana* also augmented SOD enzyme activity by 92%. All the extracts significantly increased CAT activity, reverting H_2_O_2_ effects. *D. arctica*, *V. pinastri* and *T. americana* showed the highest values (100.9%, 97% and 95%, respectively), followed by *N. stracheyi*, with moderate CAT activity (81.6%).

### 2.4. Lichen Extracts from Cetrarioid Clade Protected against H_2_O_2_-Induced Mitochondrial Dysfunction

**Figure 5:**
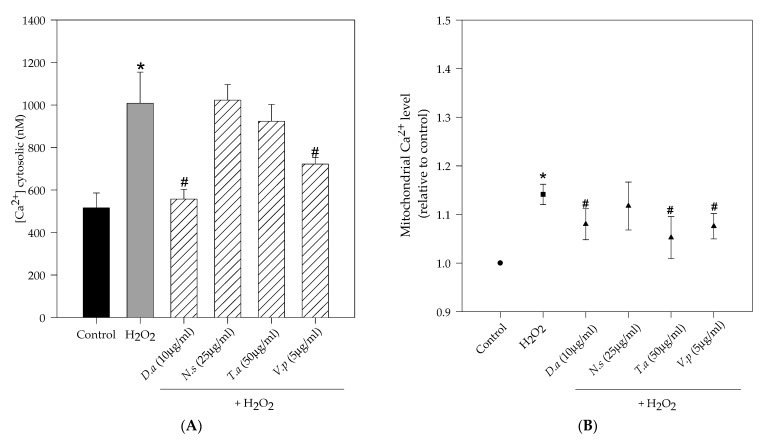

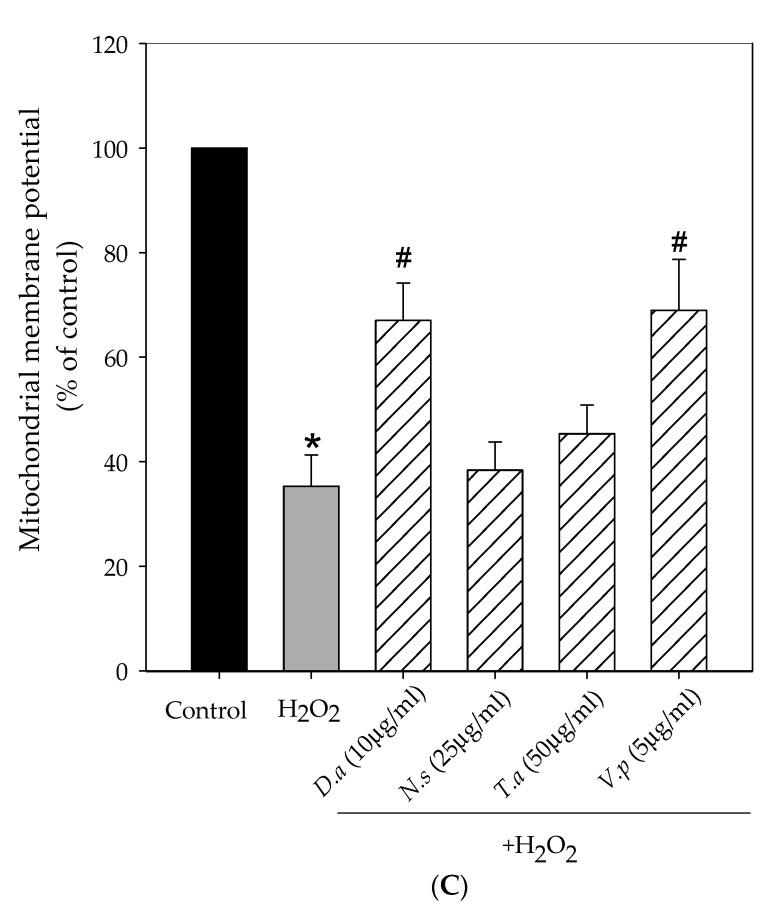
Effect of lichen extracts against H_2_O_2_ -induced mitochondrial dysfunction in SH-SY5Y. (**A**) on cytosolic calcium levels. (**B**) on mitochondrial calcium levels. (**C**) on mitochondrial membrane potential. Data are expressed as means ± SD (% of control) * *p* < 0.001 vs. control; # *p* < 0.001 vs. H_2_O_2_).

Figure 5 shows the effect of lichen extracts on different mitochondrial parameters (mitochondrial membrane potential and calcium levels). Treatment with hydrogen peroxide (250 µM, 1 h) caused a significant decrease in the mitochondrial membrane potential (35% compared to 100% control cells) and a significant increase in mitochondrial calcium levels (1.14 relative to control) and cytosolic calcium levels (1007 nM compared to 516 nM control cells). However, pretreatments with lichen extracts prevented H_2_O_2-_induced mitochondrial changes. In particular, extracts of *D. arctica* (10 µg/mL) and *V. pinastri* (5 µg/mL) significantly increased mitochondrial membrane potential by 67% and 69%, respectively, and significantly reduced cytosolic calcium levels by 557 nM and 721 nM, respectively. Mitochondrial calcium levels were reduced in pretreated cells with selected concentrations of *D. arctica*, *V. pinastri* and *T. americana* extracts (1.08, 1.07, 1.05 relative to control, respectively).

### 2.5. HPLC Profile of Lichen Extracts from Cetrarioid Clade

**Figure 6:**
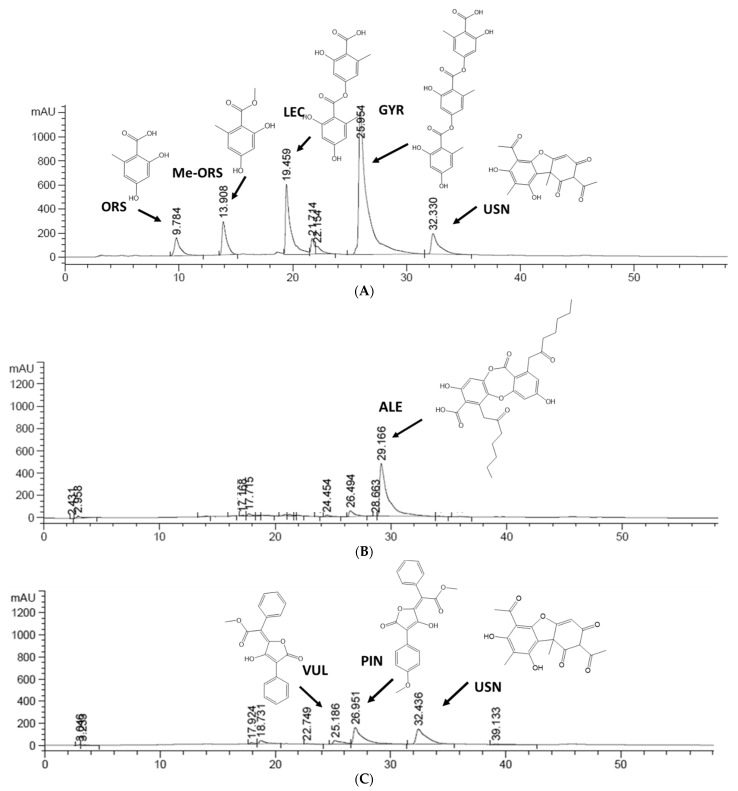
Representative HPLC chromatograms (λ = 254 nm) (**A**) *Dactylina artica* (**B**) *Tuckermannopsis americana* (**C**) *Vulpicida pinastri*.

The most promising lichen extracts were analyzed using the HPLC-UV method, whose representative chromatograms are shown in Figure 6. Secondary metabolites were identified based on their retention times and ultraviolet spectra as compared to standards and previously reported lichen extracts. Table 1 reported retention times and the absorbance maxima (nm) UV spectrum. Results showed that the main compounds in *D. artica* were gyrophoric acid (GYR) and lecanoric acid (LEC). The lichen *T. americana* contained alectoronic acid (ALE). The compounds usnic acid (USN), pinastric acid (PIN) and vulpinic acid (VUL) were the majority in *V. pinastri*.

## 3. Discussion

The present work demonstrated that pretreatments with methanol lichen extracts from cetrarioid clade provide neuroprotection against hydrogen peroxide in SH-SY5Y cells as evidenced in ROS reduction, improvement in oxidative stress biomarkers and antioxidant enzyme activity and mitochondrial protection. 

The brain is a metabolically active organ that yields high ROS levels compared to other organs. Moreover, neurons are the most sensitive cell types to free radicals [11]. Overproduction of ROS can lead to oxidative macromolecules injury and consequently, to cell death. Activation of necrotic and apoptotic pathways by ROS induces cell death. Among the mechanisms responsible for ROS-causing apoptosis are receptor activation, caspase activation and mitochondrial dysfunction [12]. Oxidative stress (ROS/antioxidant imbalance) has been implicated in the initiation and progression of age-related neurodegenerative diseases [13]. Therefore, the prevention of oxidative stress is one of the most promising strategies for all those diseases that involve an alteration of redox homeostasis. H_2_O_2_ acts as an inducer of oxidative stress damage, increasing ROS levels and leading to cell death. The current study found that methanol lichen extracts from cetrarioid clade significantly attenuated hydrogen peroxide-induced ROS production in the human neuroblastoma SH-SY5Y cell line and consequently prevented cell death. Hydrogen peroxide can cross cell membranes and cause oxygen-derived free radicals. Hence, hydrogen peroxide can be converted into hydroxyl radicals in the presence of ferrous ions (Fenton reaction) [14]. Lichens contain phenolic compounds in their composition which can act as antioxidants through mechanisms such as radical scavenging activity and metal chelating activity [15,16,17]. 

Furthermore, these methanol lichen extracts mitigated changes in biomarkers of oxidative stress (lipid peroxidation reduction and GSH increase). The brain presents the highest rate of lipid metabolism in the body. In Alzheimer’s disease and in Parkinson’s diseases, there is an overproduction of ROS that induces the oxidation of lipid membrane constituents, leading to lipid hydroperoxides. This process, named lipid peroxidation, constitutes a hallmark for most neurodegenerative disorders. In fact, polyunsaturated fatty acids (PUFAs) are the main target of ROS attack, due to the high number of double bonds in their structure [18,19]. This early event in the brain causes cytotoxic and genotoxic effects. TBARS is a common biomarker of polyunsaturated fatty acids peroxidation. High amounts of lipid peroxidation products have been identified in post-mortem brains of people affected with neurodegenerative diseases. These lipid peroxidation products cause tissue injury and failures of antioxidant systems [20]. The lichen extracts of *V. pinastri* and *D. arctica* markedly reduced lipid peroxidation in neuroblastoma cells. Glutathione (GSH) is the major endogenous antioxidant defense. This primary antioxidant scavenges free radicals through its thiol group of its cysteine residue, and it functions as a co-substrate of the antioxidant enzymes selenium-glutathione peroxidase (GPx) and glutathione S-transferase (GST) [21]. GPx reduces lipid peroxides to alcohols and aldehydes. It has been reported that a reduction of GSH to its oxidized form provokes a decrease in intracellular GSH [22]. Restoring the levels of GSH is strongly related to the ROS and lipid peroxidation reduction [18]. Previous studies reported that fumarprotocetraric acid (depsidone), evernic acid (depside) and usnic acid (dibenzofuran-like) inhibited ROS generation, lipid peroxidation and glutathione depletion in neurons and the astrocytes cell model using hydrogen peroxide as an oxidative stress inductor [23,24]. In addition, the Parmeliaceae lichens *Cetraria islandica* and *Vulpicida canadensis* also showed protective effects against H_2_O_2_-induced injury in the human astrocytoma cell line U373-MG, as evidenced by reduced ROS production, increased GSH levels and the inhibition of lipid peroxidation [9]. 

Moreover, lichen extracts increase SOD and CAT activity. The enzyme SOD catalyzes the dismutation of superoxide anion to hydrogen peroxide which is then converted into oxygen and water by the action of the antioxidant enzymes catalase and glutathione peroxidase. The enzymatic activity of SOD and CAT showed to be significantly reduced in postmortem brain tissue at an advanced age [22]. Therefore, the results of this study showed that of the four methanol lichen extracts tested, *N. stracheyi* exerted its neuroprotective activity via ROS inhibition and increased CAT activity while *V. pinastri*, *D. arctica,* and *T. americana* prevented ROS overproduction and maintained enzymes activity. Upregulation of enzymes activity could be associated with an increase in its expression. Previously, it has been demonstrated that the depside fumarprotocetraric acid, isolated from *Cetraria islandica,* upregulated the antioxidant enzymes catalase, superoxide dismutase-1, and hemoxigenase-1 expression which was related to Nrf2 signaling pathway activation [23]. Other lichens such as *Parmotrema perlatum* and *Hypotrachyna formosana* have also evidenced to reduce intracellular ROS generation, inhibit the peroxidation of lipids, and increase GSH levels and SOD activity [25].

Mitochondria are a major cellular organelle that play a key role in aging and degenerative diseases and are a target for oxidative damage. Mitochondria are the main source of ROS, particularly of superoxide radicals, through the complexes I and III of the respiratory chain [26,27]. An overproduction of mitochondria ROS may alter membrane permeability and calcium homeostasis as well as induce DNA mutations and injure the mitochondrial respiratory chain [28]. In our study, methanol lichen extracts of *V. pinastri* and *D. arctica* prevented mitochondrial changes by regulating calcium homeostasis and increasing mitochondrial membrane potential, suggesting a protective activity against H_2_O_2_. These extracts which target mitochondria are of great interest because they can pass across the mitochondrial phospholipid bilayer and reduce ROS damage at the heart of the source [29]. Other studies demonstrated that the depsidone fumarprotocetraric isolated from *Cetraria islandica* prevented mitochondrial membrane potential dissipation and mitochondrial calcium increase [9].

The analytical study by HPLC-UV revealed that the major compounds presented in *V. pinastri* were usnic acid, pinastric acid and vulpinic acid, and in *D. arctica* were gyrophoric acid, lecanoric acid and usnic acid, while in *T. americana* it was alectoronic acid. Lichen compounds are biosynthesized through three pathways: via the acetylpolymanolate pathway which produces depsides, depsidones and dibenzofurans, the shikimic pathway which produces pulvinic acids and the mevalonic acid pathway which is involved in terpenes formation. Therefore, gyrophoric acid and lecanoric acid are depsides, usnic acid is a dibenzofuran, alectoronic acid is a depsidone and vulpinic acid and pinastric acid are pulvinic acids [30,31]. All these lichen compounds have shown a great diversity of activities including anti-cancer (i.e., gyrophoric acid, vulpinic acid), antimicrobial (i.e., gyrophoric acid, usnic acid, vulpinic acid) photoprotective (i.e., gyrophoric acid) and neuroprotective (i.e., usnic acid) activities [32,33,34,35,36,37]. 

Based on the chemical structure of lichen compounds and its potential antioxidant activity, the depsides gyrophoric acid and lecanoric acid have carboxyl and hydroxyl groups that interact with several enzymatic active sites. Moreover, the aromatic rings of gyrophoric acid and lecanoric acid are responsible for their free radical scavenging properties [38,39]. Among the lichens investigated in this study, *D. artica* was the most active specie. Previous works have shown that *D. artica* has potent antioxidant properties (ORAC value 8.2 μmol TE/mg dry extract, DPPH value IC_50_ 346.3 μg/mL and FRAP value 29.6 μmol of Fe^2+^ eq/g sample) which are attributed to the anti-free radical properties of gyrophoric acid and lecanoric acid [10].

On the other hand, the antioxidant properties of *V. pinastri* are mainly due to the presence of vulpinic acid and pinastric acid. These pulvinic acids have a butanolide ring with an -OH group at the 4-position and a carboxylic acid function at the double bond. This double bond is involved in radical stabilization, and is a good descriptor of antioxidant properties [17].The antioxidant activity of pulvinic acids has been demonstrated using quantitative structure–activity relationship (QSAR) techniques combined with a multivariate analysis [40,41].

Regarding lichen compounds with a depsidone structure, previous studies revealed that they are potent hydroxyl and superoxide anion radical scavengers in polar environments but not good peroxyl radical scavengers [15]. Moreover, a better hydrogen-donating potency in those depsidones with no butyrolactone ring has been reported; this is the case of alectoronic acid, which has been identified in *T. americana* [42]. Finally, the compound usnic acid, presented in *D. arctica* and *V. pinastri*, has shown reducing potential in DPPH, ABTS and DMPD radical cation assays, and superoxide radical and peroxyl scavenging abilities [16,37]. In addition to this, the presence of a phenolic ring with functional groups of −CO, −COH and −COOH showed metal chelating ability, including Fe^2+^ ion [16].

## 4. Materials and Methods

### 4.1. Reagents

All reagents were acquired from Sigma-Aldrich (St. Louis, MO, USA) except for HPLC grade methanol and dimethyl sulfoxide (DMSO), that were purchased from Panreac (Barcelona, Spain). Molecular probes were obtained from Invitrogen-Thermo Fisher Scientific (Carlsbad, CA, USA).

### 4.2. Lichen Collection and Preparation of Methanol Extracts

The lichens *Dactylina arctica* (Central Siberia, Russia, July 1995; MAF-Lich 96262), *Nephromopsis stracheyi* (North Sikkim, India, August 2004; MAF-Lich 22748), *Tuckermannopsis americana* (Maine, USA, June 2010; MAF-Lich 19828) and *Vulpicida pinastri* (Alto del Peñón, Zamora, Spain, September 2017; MAF-Lich 22753) were identified and authenticated by Dr. P.K. Divakar and Professor A. Crespo and preserved in the Herbarium of the Faculty of Pharmacy (MAF), University Complutense of Madrid (Spain).

For the preparation of extracts, 2 mL of pure methanol was mixed with dry thalli samples (50 mg) and after being shaken for 20 s, every 15 min for 2 h, was left overnight. Methanol extracts were filtered (0.45 μm pore) and evaporated at room temperature. Dry residues were stored until their use.

### 4.3. Human Neuroblastoma Cell Line (SH-SY5Y Cells)

SH-SY5Y cells were grown in DMEM supplemented with 10% fetal bovine serum and 0.5% gentamicin at 37 °C and 5% CO_2_/95% air. Confluence was between 80 and 90%.

### 4.4. Cell Treatments

Lichen extracts were dissolved in DMSO and PBS (1 mg/mL) as stock. Serial dilutions were then made with PBS. SH-SY5Y cells were pretreated with different concentrations of methanol lichen extracts for 24 h, before H_2_O_2_ (250 µM, 1 h). Final DMSO concentration was lower than 0.1% at the highest concentration. 

### 4.5. Metabolic Activity Measurement 

Survival rate and cytoprotection were determined using an MTT assay according to the method described by Mosmann [43] with some modifications. After treatments, a solution of MTT (2 mg/mL, 100 μL) was added to wells, and plates were incubated for 1 h. Then, the medium was removed, and formazan crystals were dissolved with DMSO (100 µL). Absorbance was measured at 550 nm with a Spectrostar BMG microplate reader.

### 4.6. Intracellular ROS Production 

Intracellular ROS production was determined using a DCFH-DA assay as described by LeBel et al. (1992) [44]. Briefly, DCFH-DA dissolved in DMEM medium (1%) without phenol red was added to 96-well plates for 30 min. This solution was then removed, and cells were treated with non-cytotoxic lichen concentrations for 24 h before hydrogen peroxide (250 μM). Fluorescence was measured with a microplate reader (FLUOstar OPTIMA, BMG Labtech, Ortenberg, Germany) at excitation/emission wavelength 485/528 nm.

### 4.7. BCA Assay

The protein concentration was calculated using a bicinchoninic acid (BCA) assay. The colorimetric reaction was measured at 550 nm in a Spectrostar microplate reader (BMG Labtech, Ortenberg, Germany). Samples of total cellular extracts were mixed with a reaction solution [bicinchoninic acid and copper (II) sulfate]. The purple color proportionally increased with the amount of protein. A bovine serum albumin (BSA) curve was used to normalize protein [45].

### 4.8. Glutathione Levels 

GSH content was determined following the Hissin and Hilf (1976) method with some modifications [46]. In 96-well plates, phosphate-EDTA buffer (pH 8.0, 150 µL) was mixed with total extract samples (50 µL). Then, O-phthalaldehyde (OPT) was added (20 µL) and samples were incubated for 15 min in the dark. Fluorescence was measured at an excitation/emission wavelength 360/460 nm. A standard curve of reduced GSH was used.

### 4.9. Antioxidant Enzymatic Activity

#### 4.9.1. SOD Enzymatic Activity

Superoxide dismutase catalyzes the conversion of superoxide anions into oxygen and hydrogen peroxide. In 96-well plates, it was added to the reaction mixture consisting of total cellular extracts, EDTA, buffer phosphate (pH 7.8, with 0.2% Triton X-100), hydroxylamine chlorohydrate, and nitroblue tetrazolium (NBT). NBT reduction was measured at 530 nm each minute, during 15 min, using a SPECTROstar Omega microplate reader (BMG Labtech, Ortenberg, Germany) [47]. 

#### 4.9.2. CAT Activity

In 96-well plates, total cell extracts were mixed with hydrogen peroxide (14 mM). Absorbance was measured at 240 nm wavelength for 1 min using a SPECTROstar Omega microplate reader (BMG Labtech, Ortenberg, Germany) following Aebi et al.’s method with some modifications [48].

### 4.10. TBARS Assay

Lipid peroxidation was determined by performing a TBARS assay [49]. After treatments, cell pellets were stored at 80 °C. On the day of the experiment, pellets were defrosted at room temperature and were mixed with TBA-TCA-HCl. This mixture was boiled at 100 °C for 10 min. Samples were placed on ice to stop the reaction. Then, samples were centrifuged at 4 °C (3000 rpm, 10 min) and supernatants were added into 96-well plates to measure absorbance at 530 nm using a SPECTROstar Omega microplate reader (BMG Labtech, Ortenberg, Germany). Results were expressed as a percentage of TBARS (100% of control).

### 4.11. Calcium Cytosolic Quantification

Calcium cytosolic was quantified using Indo-1/AM as a cell-permeant dye [50]. After treatments, a Krebs medium containing Indo-1/AM dye (3 mM) and calcium (1mM CaCl_2_) was added to cells for 45 min at 37 °C. Then, the medium was removed, and cells were incubated with a dye-free Krebs medium for 15 min at 37 °C in the dark. Fluorescence was recorded in a microplate reader (FLUOstar OPTIMA, BMG Labtech, Ortenberg, Germany) at 350 nm excitation wavelength and at 410 nm emission wavelength. The formula for cytosolic calcium concentration was [Ca^2+^]_i_ =Kd × [F−Fmin]/[Fmax−F], where Kd is the dissociation constant for Indo-1; F is the fluorescence signal for samples; Fmax is the maximum fluorescence signal after ionomycin addition and *Fmin* is calculated using this formula: *Fmin* = AF + 1/12 × (*Fmax*-AF), AF being the minimum fluorescence after adding MnCl_2._

### 4.12. Mitochondrial Calcium Quantification

Mitochondrial calcium was quantified using Rhod-2/AM as a fluorescent cationic probe [51]. After treatments, cells were incubated in a Krebs medium containing 0.1% BSA, 1 mM calcium and 10 mM Rhod-2/AM during 40 min at 37 °C. Then, cells were maintained in dye-free Krebs medium cells in this medium for 30 min at 37 °C in the dark. Basal fluorescence intensity was recorded using a microplate reader (FLUOstar OPTIMA, BMG Labtech, Ortenberg, Germany) at λ 552 nm excitation wavelength and λ 581 nm emission wavelength for 5 min at 37 °C. Then, maximum fluorescence was measured for 15 min after adding calcium ionophore A23187 (5 μM) in the same conditions as described above. Mitochondrial calcium levels were the ratio between fluorescence measures before and after ionophore addition. Results were expressed relative to control.

### 4.13. Mitochondrial Membrane Potential (MMP)

Mitochondrial membrane potential was quantified using the fluorescent cationic dye tetramethylrhodaminemethylester (TMRM) following Correia et al.’s (2012) protocol with some modifications [52]. After cell treatments, the Krebs medium with calcium (1 mM CaCl_2_) and TMRM (250 nM) was added. A FLUOstar OPTIMA (BMG Labtech, Ortenberg, Germany) microplate reader was used to measure basal fluorescence activity at λ 549 nm excitation and λ 573 nm emission at 37 °C for 45 min. Then, the maximum fluorescence value was estimated after adding FCCP (6 mM) and oligomycin (0.25 mg/mL). Mitochondrial membrane potential (Δψm) is the result by subtracting fluorescence basal values from maximum fluorescence values. Results were expressed as % of the control. 

### 4.14. Secondary Metabolites Detection Using High Performance Liquid Chromatography

HPLC analysis was used for secondary metabolites detection using the method described by de Paz et al. (2010) [53]. The HPLC instrumentation was an Agilent 1260 instrument (Agilent Technologies, CA, USA) equipped with a photodiode array detector (190–800 nm) and a reversed-phase Mediterranean Sea 18 column (150 mm × 4.6 mm, 3 µm particle size; Teknokroma, Barcelona, Spain). Running conditions included: a mobile gradient phase [1% orthophosphoric acid in milli-Q water (A)/methanol (B)]; a flow rate of 0.6 mL/min; a column temperature of 40 °C and a UV spectrum between 190 and 400 nm. Secondary metabolites of lichens were identified by comparing the retention time and UV absorption spectra with standard compounds (commercialized and isolated previously by our research team) and other lichen species [9,54]. 

### 4.15. Statistical Analysis

All assays were measured in triplicate and data were expressed as mean ± SD. Statistical analysis was performed by SigmaPlot 11.0 using analysis of variance (ANOVA) and Tukey’s post hoc test (5% significance level).

## 5. Conclusions

In conclusion, our findings provide evidence of the protective activity of methanol extracts obtained from cetrarioid clade against the neurotoxic effects of hydrogen peroxide in neuroblastoma cells. *D. arctica* and *V. pinastri* afford the highest protective effect. Future research should be aimed at studying the protective activity of isolated compounds from *D. arctica* and *V. pinastri*, delving into their mechanism of action. 

## Figures and Tables

**Table 1:** Retention times and UV absorbance maxima (nm) of main secondary metabolites of studied lichens.

Compounds	Retention Time (t_R_, min)	UV Detection (λ max, nm)
Alectoronic acid (ALE)	29.1 ± 0.001	214, 254, 316
Gyrophoric acid (GYR)	25.9 ± 0.003	212, 270, 304
Lecanoric acid (LEC)	19.4 ± 0.0312	212, 270, 304
Methyl orsellinate (Me-ORS)	13.9 ± 0.002	210, 262, 298
Orsellinic acid (ORS)	9.8 ± 0.011	210, 262, 298
Pinastric acid (PIN)	26.9 ± 0.047	<210, 246, 392
Usnic acid (USN)	32.3 ± 0.046	226/234, 282
Vulpinic acid (VUL)	25.1 ± 0.034	<210, 234, 282, 354

## Data Availability

Not applicable.

## Sample Availability

Samples are not available from the authors.
